# LHO-net: A Lightweight Steel Defect Detection Framework Based on Cross-Scale Feature Selection and Adaptive Optimization

**DOI:** 10.3390/s26061990

**Published:** 2026-03-23

**Authors:** Qi Wang, Haocheng Yan

**Affiliations:** School of Computer Science, Nanjing University of Information Science and Technology, No.6 Ningliu Road, Nanjing 210044, China; 002086@nuist.edu.cn

**Keywords:** defect detection, lightweight detection model, multi-level feature fusion, cross-scale feature fusion

## Abstract

To address the issues of poor adaptability to complex scenarios, high computational complexity, and difficulties in terminal deployment of existing steel surface defect detection models, a novel lightweight detection network named LHO-net is proposed, with the Lightweight Multi-Backbone (LM Backbone), the Hierarchical Scale-based Pyramid Attention Network (HSPAN), and the Occlusion-aware Detection Head (OAHead). The LM Backbone adopts a dual-branch structure with shared HGStem and a dynamic feature fusion mechanism, effectively capturing multi-dimensional features of irregular defects while extremely compressing model parameters. The HSPAN module realizes efficient fusion of multi-scale features through dynamic feature selection and adaptive upsampling strategies, balancing background noise suppression and defect detail preservation. The OAHead completes adaptive compensation of features in occluded regions by means of deep feature aggregation and exponential normalization technology, significantly enhancing the ability to recognize complex defects. On the NEU-DET dataset, LHO-net achieves a mAP@0.5 of 75.0%, a mAP@0.5:0.95 of 44.0%, and a recall of 73.6%, with a computational complexity of only 2.3 GFLOPS. Compared with the baseline model YOLOv12, it reduces parameters by 64% and computational cost by 60.3%. On the GC-10 dataset, its mAP@0.5 reaches 67.2%, and its detection stability for complex defects such as slender creases and low-contrast water spots is superior to that of mainstream lightweight YOLO variants. Visualization results confirm that the model can effectively avoid common problems such as redundant annotations and false detections and maintains stable recognition performance for various defects. It solves the core contradiction between detection accuracy and lightweight deployment in industrial scenarios, providing an efficient and practical technical solution for real-time steel surface defect detection on resource-constrained terminal devices.

## 1. Introduction

Over the past decades, steel materials, as fundamental raw materials, have been extensively applied in fields such as transportation, industrial manufacturing, national defense, and construction, playing a pivotal supporting role in the industrialization process [1,2]. However, during complex processes such as production, storage, transportation, and subsequent processing, steel surfaces are highly susceptible to various defects caused by factors like collisions, scratches, and impurity inclusion. These defects not only directly impair the performance and lifespan of end products but also significantly reduce the product qualification rate for manufacturers, thereby increasing quality-related costs. Consequently, developing efficient and precise automated defect detection technologies for steel surfaces is of substantial practical significance for ensuring product quality, enhancing production efficiency, and safeguarding operational safety [3].

Currently, steel surface defect detection is still predominantly performed through manual visual inspection. However, this method is characterized by strong subjectivity, low efficiency, and inconsistent judgment criteria, making it increasingly inadequate to meet the demands for high speed, consistency, and accuracy in the inspection process imposed by modern production lines [4]. Consequently, the realization of rapid, precise, automated, and intelligent defect identification has become a critical pathway through which labor costs can be effectively reduced, product quality can be ensured, and both production efficiency and core competitiveness can be enhanced.

From a technological evolution perspective, vision-based steel surface defect recognition methods are considered to have undergone three major stages: manual inspection, traditional image processing, and deep learning [5,6]. Before the mid-20th century, manual visual inspection was adopted as a feasible quality control method, being limited by the technical conditions and production scale of the time. However, this method was entirely reliant on the experience and on-site judgment of inspectors and was characterized by inherent drawbacks such as significant subjective bias, high cost, and a tendency for fatigue-induced missed inspections, rendering it incompatible with today’s high-speed automated production environments [7].

With the development of computer technology, defect detection methods based on digital image processing techniques began to be developed. The fundamental principle is that defect areas are typically differentiated from normal areas by shallow visual features such as grayscale, texture, or color. Through the design of specific image processing algorithms, the localization and preliminary identification of defects are achieved. However, the core feature extraction and judgment rules in such methods are heavily dependent on manual prior design and parameter tuning, resulting in limited generalization capability. Once changes in the production environment or defect morphology occur, the detection performance is prone to a sharp decline [8].

In recent years, deep learning-based detection methods have gradually emerged to address these shortcomings of traditional approaches. Currently, deep learning-based detection methods are categorized into two types: one-stage detection algorithms and two-stage detection algorithms. One-stage detection algorithms, represented by SSD [9], RetinaNet [10] and the YOLO (You Only Look Once) series [11], treat object detection as a regression problem. The bounding box positions and class probabilities are directly output from the entire image through a convolutional neural network (CNN). The primary advantage of such one-stage algorithms lies in their extremely fast detection speed, enabling the processing of large volumes of image data within short timeframes. Consequently, they hold irreplaceable application value in scenarios requiring high real-time detection capabilities, such as rapid defect inspection on industrial production lines and real-time object monitoring in intelligent transportation systems. Yu et al. [12] proposed a detection network based on the YOLOv12s architecture, which is equipped with a Separated and Enhancement Attention Module (SEAM) as the core to isolate occluded regions and enhance the representation of valid defect features for occluded defect detection, a Receptive Field Enhancement (RFE) module for auxiliary multi-scale feature extraction, and a Slide Weight Function (SWF) to mitigate the imbalance between hard occluded and easy unoccluded defect samples. Hu et al. [13] proposed the lightweight fusion network DFFNet for fast and accurate steel surface defect detection, which is equipped with a lightweight backbone network LDD that utilizes partial convolution to reduce computational complexity and extract spatial features efficiently, enhanced PANet integrated with the Efficient Feature-Optimized Converged Network and a Feature Enhancement Aggregation Module (FEAM) to improve feature fusion to extend the receptive field for defect perception and reduce information loss for small defects. Lu et al. [14] proposed WSS-YOLO which uses WIoU loss-based dynamic focusing, C2f-DSC (Dynamic Snake Convolution), and GSConv/VOV-GSCSP modules to boost industrial steel defect detection performance while reducing model complexity. Extensive experiments on NEU-DET and GC-10 datasets show the model achieves mAP of 82.3% and 72.0%, respectively, outperforming other excellent models and validating its effectiveness in industrial defect detection.

The other category is represented by two-stage detection algorithms such as Faster R-CNN [15] and Mask R-CNN [16]. In the first stage, candidate regions are generated, while in the second stage, the candidate regions produced in the first stage are classified and subjected to precise bounding box regression through convolutional operations. Ren et al. [17] proposed an improved Faster R-CNN algorithm by replacing the convolutional layers for feature extraction with depthwise separable convolutions and incorporating center loss into the original loss function to enhance the network’s ability to distinguish between different types of defects. This algorithm achieved a detection accuracy of 98.32%. Shi et al. [18] built upon Faster R-CNN by adopting the emerging ConvNeXt architecture as the backbone for feature extraction in Faster R-CNN. They also introduced the CBAM attention module and the K-means clustering algorithm to improve the model’s generalization capability, meeting the real-time demands of industrial inspection. Zhu et al. [19] introduced an anchor parameter adjustment method based on K-means clustering to effectively detect slender defects. To improve the segmentation performance of image patches by eliminating holes and edge burrs, the Felzenszwalb algorithm was applied to optimize the results.

The aforementioned methods have proven effective in enhancing accuracy. However, due to the real-time requirements of actual factory production, the defect detection process must not only ensure precision but also be capable of online real-time analysis and monitoring. Currently, many studies achieve high-accuracy detection by employing highly complex network structures. Nevertheless, these sizable models pose deployment challenges on factory equipment with limited computational resources and require longer response times for algorithm execution. Balancing detection accuracy with model lightweighting represents a significant challenge in applying defect detection to practical engineering fields. Achieving high detection accuracy while satisfying real-time performance demands remains a formidable task.

To address the limitations of existing steel surface defect detection models, this study focuses on the steel surface defect detection task and proposes LHO-net, a lightweight detection framework based on cross-scale feature selection and adaptive optimization:

(1) A Lightweight Multi-Backbone (LM Backbone) with a shared Hierarchical Group Stem (HGStem) and dynamic feature fusion mechanism. This dual-branch architecture integrates an improved HGNetV2 and a C2f-based branch, synergistically capturing multi-dimensional features of irregular steel defects while achieving extreme compression of model parameters and computational overhead.
(2) A Hierarchical Scale-based Pyramid Attention Network (HSPAN) that realizes efficient multi-scale feature fusion through dynamic feature selection and adaptive upsampling strategies. By leveraging high-level semantic information to guide the screening of low-level detailed features, it effectively addresses the challenge of significant scale variation in steel defects.
(3) An occlusion-aware detection head (OAHead) featuring deep feature aggregation and exponential normalization technology. This module adaptively compensates for features in occluded regions while enhancing effective features in non-occluded areas, significantly improving the model’s ability to recognize complex occlusion defects.

## 2. Related Work

Currently, deep learning-based defect detection methods are often too complex and require substantial computational resources, making them unsuitable for deployment on terminal devices with limited computational capacity. To address this issue, Zhang et al. [20] proposed an efficient detection model incorporating a dual-backbone architecture with hierarchical attention fusion. The model employs DualFuseNet as its backbone, which utilizes a dual-path structure based on CSPDarknet and HGNetV2 to achieve collaborative extraction and weighted fusion of multi-scale features via the HiAFusion module. Additionally, a lightweight hybrid encoder is introduced, integrating an efficient upsampling module and a heterogeneous convolutional branch optimization mechanism. This design significantly reduces computational complexity while maintaining multi-scale fusion capability, effectively improving the accuracy of berry detection. With only 0.38 M parameters, 1.4 G FLOPs computational load, and a model size of 1.2 MB, the proposed model is well-suited for deployment on unmanned aerial vehicles or edge computing platforms. Zhang et al. [21] proposed a lightweight object detection model based on YOLOv5s. The model designs a lightweight asymmetric detection head to replace the original structure and introduces a C3CA module incorporating a coordinate attention mechanism. In the backbone network, the C3 module is replaced with a FasterConv module, and semantically similar features are effectively reconstructed through a content-aware feature reassembly module. Additionally, a novel XIoU loss function is proposed to enhance the model’s convergence speed and robustness. Hu et al. [22] proposed CCDFormer, a dual-backbone U-shaped network based on Transformer. The model independently extracts multi-view features through dual-path architecture, incorporates deformable convolution to adapt to crack morphology, and designs feature enhancement modules along with pyramid Transformers to respectively strengthen multi-scale local features and model long-range dependencies. Finally, a feature fusion module generates robust crack representations. Experiments on public datasets demonstrate that the model outperforms existing methods in key metrics such as accuracy and recall, effectively meeting the requirements for crack detection in real-world scenarios. Peng et al. [23] proposed the YOLOv7-SGS model, which integrates the Shape-IoU, SGE attention mechanism and GSConv algorithm. It achieves a 6% absolute improvement in mAP@0.5 while enabling real-time detection at 32 FPS, thus providing an efficient solution for industrial applications. Tie et al. [24] proposed a lightweight steel surface defect detection model based on an improved YOLOv8n framework. By introducing Kernel-aware Convolution (KWConv) and a Bidirectional Feature Pyramid Network (BiFPN), the model reduces both computational load and parameter count while enhancing multi-scale feature fusion capabilities. Furthermore, the model incorporates a Receptive Field Block (RFB) and a Large Kernel Separable Attention (LSKAttention) mechanism to effectively expand the receptive field and strengthen target feature representation. This approach achieves higher detection accuracy with reduced resource consumption. Chen et al. [25] proposed the MFDS-DETR model, which boosts representation capability for multi-scale white blood cells via a High-level Screening Feature Fusion Pyramid. This module adopts a channel attention mechanism to screen and fuse multi-level features with high-level feature weights. The encoder incorporates a multi-scale deformable self-attention module, and the decoder integrates self-attention with cross-deformable attention, thereby enabling effective extraction of global features from white blood cell images. Wang et al. [26] proposed MFP-YOLO for lightweight UAV aerial imagery detection, replacing YOLOv5s’ original head with a lightweight decoupled detection head. This design effectively accelerates network convergence and strengthens multi-scale target detection capability under complex backgrounds. Experimental results on the VisDrone 2019 dataset show the model achieves a 79.2% reduction in parameter volume while improving the mAP50 metric significantly, validating the efficiency of the lightweight decoupled head in balancing model compactness and detection performance. Zhao et al. [27] proposed RSLH-YOLO for personnel and equipment detection in open pit mines based on YOLOv11, which introduces receptive field attention convolution and dilated convolution into the backbone to expand the receptive field and improve localization capability, adopts a high-low resolution feature bidirectional fusion mechanism with a small-target detection layer to enhance multi-scale recognition, and designs a lightweight detection head to reduce model parameters and computational costs while improving occlusion handling performance. Experiments show the model achieves an mAP of 89.1%, 3.2 percentage points higher than the baseline, and maintains high detection efficiency.

## 3. Method

### 3.1. Structure of LHO-net

**Figure 1:**
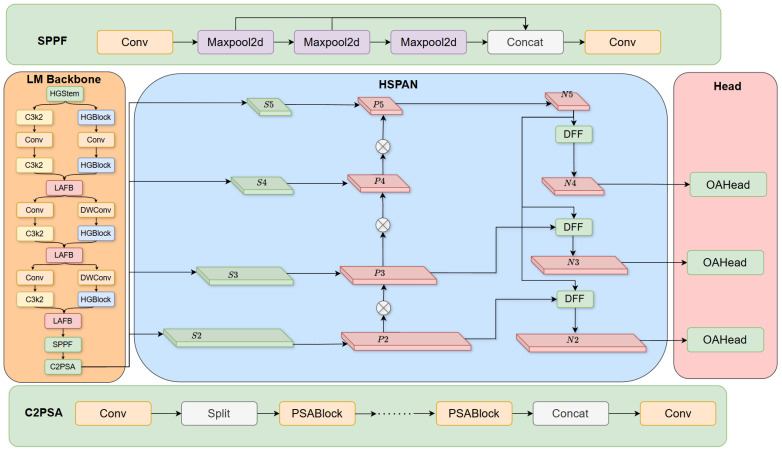
Structure of LHO-net.

The overall structure of this LHO-net, as shown in Figure 1, consists of three main components: a Lightweight Multi-Backbone network, a Hierarchical Scale-based Pyramid Attention Network, and an Occlusion-Aware detection head. With lightweight design as the core principle, the model targets the issues of feature loss and boundary blurring caused by oxide scale coverage, oil stain adhesion, and dense distribution occlusion of steel defects while controlling the number of parameters and computational complexity, thus achieving accurate localization and recognition of defects under complex working conditions.

The LM Backbone network serves as the core unit for feature extraction in the model, responsible for efficiently capturing multi-scale defect features. Taking a lightweight basic network as the framework, this module uses HGStem as the initial feature extraction unit, constructs the basic feature extraction chain through the alternating stacking of C3k2 modules and HGBlocks, embeds Layer Attention Fusion Block (LAFB) and combines it with Depthwise Convolution (DWConv) to further reduce the number of computation parameters while enhancing the correlation of features in channel and spatial dimensions; at the end, it connects Spatial Pyramid Pooling Fast (SPPF) in series to realize multi-scale spatial information aggregation, cooperates with C2f with Parallel Spatial Attention (C2PSA) via convolution, feature splitting, multi-round PSABlock attention operations and feature concatenation processes to enhance the global correlation of features, and finally outputs a series of multi-scale feature maps S2–S5. This design effectively reduces the computational complexity of the backbone network while ensuring feature expression capability, making it suitable for real-time detection requirements in industrial scenarios.

The HSPAN is designed with a low-complexity fusion strategy for the lightweight features output by the backbone network, while compensating for the feature loss in occluded regions. This module receives the S2–S5 feature maps, adopts an adaptive upsampling and direct feature fusion mechanism to replace the traditional complex convolution fusion architecture, reducing intermediate computation links; introduces a lightweight feature refinement module; and repairs the feature loss caused by occlusion through local feature enhancement and cross-level information complementation. Under the constraint of lightweight design, HSPAN realizes deep fusion of multi-scale features and finally outputs optimized feature maps N2–N5, providing high-quality feature support for subsequent detection tasks.

The OAHead adopts a lightweight detection architecture, which is adapted to the lightweight characteristics of the front-end features. Based on the N2–N5 feature maps output by HSPAN, this module integrates an occlusion-aware attention mechanism and completes the adaptive compensation of features in occluded regions and the enhancement of effective features in non-occluded regions through serial operations of feature deep aggregation, exponential normalization, and adaptive weight assignment; it realizes precise regulation of feature responses by element-wise multiplication of attention weights and the original feature maps.

### 3.2. Lightweight Multi-Backbone Network

Currently, mainstream single-stage models such as YOLOv12 adopt a core backbone architecture composed of regional attention modules, Residual Efficient Layer Aggregation Network and lightweight convolutions. Although a balance between attention-based feature extraction and real-time performance has been achieved in general object detection tasks, obvious inadequacies are still exhibited when addressing the key challenge of detecting irregular patterns in steel defect detection. Irregular steel defects typically lack fixed morphologies, are distributed across multiple regions, and feature distorted and blurred edges. However, the localized segmentation design of the regional attention module leads to the fragmentation of the global feature correlation of such defects. The combination of R-ELAN and fixed convolution kernels lacks flexible spatial transformation capabilities, making it difficult for adaptive capture of the complex morphologies and topological structures of defects. Meanwhile, its multi-scale feature fusion relies on fixed weights and hierarchical aggregation, without an effective fusion mechanism tailored to the scale heterogeneity of irregular defects. These limitations highlight the necessity of a more advanced architecture, through which efficient extraction and fusion of multi-scale features of irregular defects can be realized, and the detection performance can be improved.

**Figure 2:**
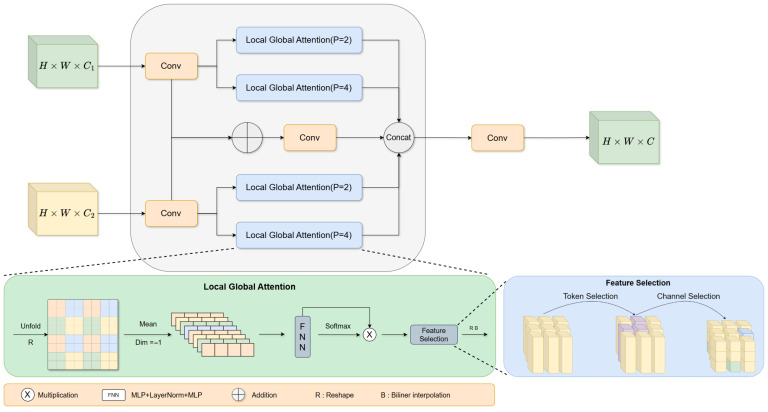
Structure of Lightweight Multibackbone.

**Figure 3:**
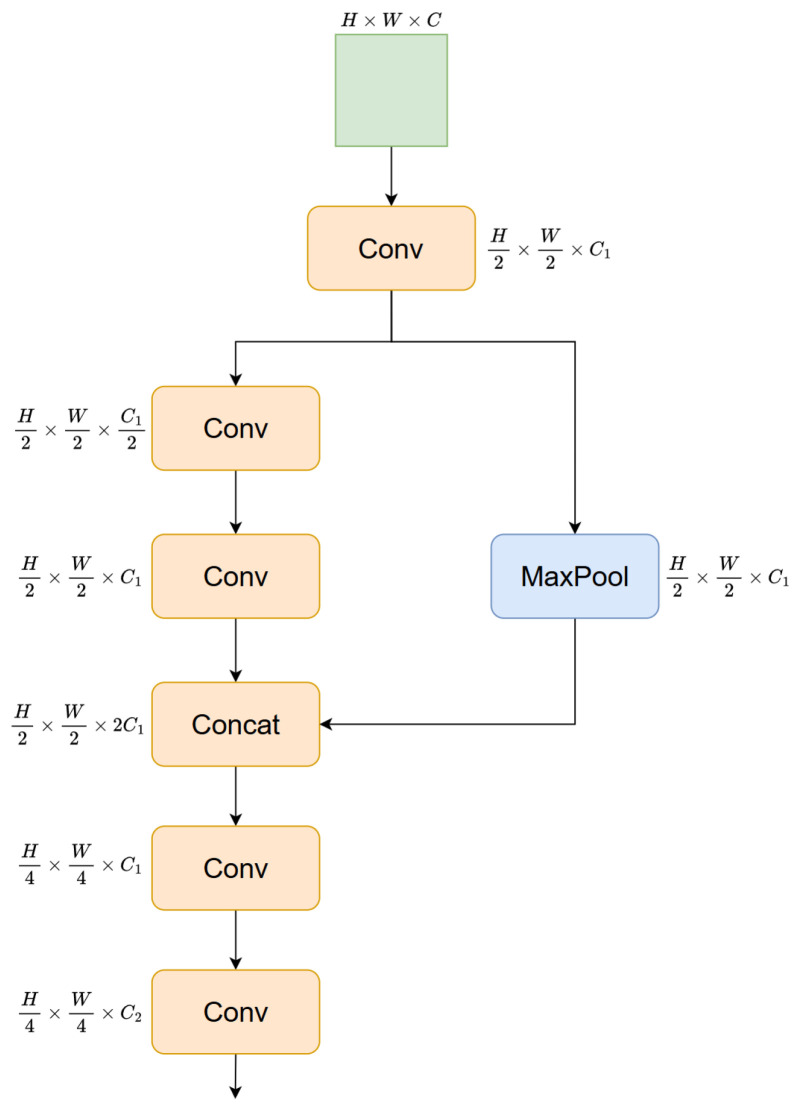
Structure of HGStem.

To address the aforementioned challenges, a Lightweight Multi-Backbone (LM Backbone) network architecture with adaptive cross-fusion capability is proposed, as illustrated in Figure 2. Its core design concept revolves around three key objectives: efficient feature extraction, redundant computation suppression, and multi-dimensional information fusion. Through the collaborative work of two functionally complementary lightweight backbone branches, the ability to capture and discriminate features of irregular defects is comprehensively enhanced. Specifically, the architecture adopts an improved HGNetv2 and a C2f structure-based branch as dual feature extraction pathways, providing support for mining the morphological, textural, and semantic information of steel defects from different dimensions. To minimize redundant computation during shallow feature extraction to the greatest extent, the two branches innovatively share a common backbone module—HGStem, as illustrated in Figure 3. This design not only significantly improves the inference efficiency of the model but also fundamentally avoids the repetitive computation problem caused by independent backbones in traditional dual-branch architectures.

The HGStem module is deeply inspired by the efficient design philosophy of HGNetV2, as illustrated in Figure 3. As a unified input gateway for the entire Dual-Backbone network, it is entrusted with the key responsibility of standardizing the input feature format and extracting basic shallow features. Internally, through a series of well-designed and coherent operations including convolution, pooling, and feature fusion, the effective capture of low-level edge and texture patterns in input images is achieved. The backbone employs a stepwise convolution strategy to progressively strengthen feature expression capacity, thereby guaranteeing the richness of base features. Meanwhile, the side branch precisely preserves critical information via max pooling operations, effectively mitigating the loss of valuable feature details. After completing the feature extraction of both the main and side branches, the module further compresses and optimizes the fused features, ultimately achieving a perfect balance between feature richness and computational efficiency. Of particular importance is that the low-level edge and texture patterns captured by shallow convolutions are highly redundant among different backbone networks. If an independent backbone is equipped for each branch, basic operations such as 3 × 3 convolution, pooling, and downsampling will be repeatedly executed multiple times. In contrast, the HGStem only needs to perform early processing once, and the output basic features can be broadcasted to the two backbone networks as a shared feature foundation, which significantly reduces the overall computational overhead.

The first branch in the backbone is constructed based on HGBlock modules, with the core goal of enhancing contextual modeling capability during feature extraction to better capture the global correlations and local details of irregular defects. Each HGBlock module mainly comprises a lightweight depthwise convolution (DWConv) [28] and an Efficient Channel Attention (ECA) [29] module and optional shortcut connections. While ensuring computational efficiency, it maximizes the feature expression capability. To realize the gradual extraction and deepening of multi-scale semantic features, this branch adopts a differentiated HGBlocks stacking strategy in different stages. The finest granularity stage extracts subtle surface textures and low-level structural patterns of steel through two cascaded HGBlocks. This establishes a critical foundation for early defect localization. The intermediate stage employs three HGBlocks in an adaptive configuration. The first block omits shortcut connections to enforce aggressive feature compression and abstraction. The subsequent two blocks retain residual pathways to preserve information flow and support feature reconstruction, thus striking a balance between high-level abstraction and fine-detail retention. The coarsest stage deploys a single HGBlock to model high-level semantic features. This enhances the network’s ability to distinguish defect types while strictly constraining parameter count and mitigating overfitting. By utilizing depthwise convolution for spatial downsampling, the design achieves substantial reductions in computational complexity and memory usage relative to standard convolution, without compromising downsampling performance.

The second branch adopts the classic and mature C2f structure. Through the design of alternately using convolutional layers and CSP bottleneck modules, it steadily improves the feature representation capability while maintaining a lightweight computational burden throughout. The core design philosophy of this pathway emphasizes structural simplicity and deployment adaptability. Efficient feature extraction can be achieved without complex module stacking, making it highly suitable for deployment on resource-constrained devices commonly found in industrial scenarios. This characteristic provides significant convenience for the engineering application of the model, enabling it to adapt to the practical operational requirements of industrial environments where computing resources may be limited.

Visual detection tasks generally rely on multi-scale feature fusion and attention mechanisms to enhance the model’s capability of perceiving complex scenarios. However, mainstream methods currently face difficulties in establishing effective correlations between local structural details and global semantic information during the fusion of multi-source heterogeneous features. This deficiency causes the generated features to lack the support of critical details, thereby resulting in insufficient representational capacity and weak discriminative performance. To fully leverage the complementary advantages of the two backbone branches and achieve a “1 + 1 > 2” feature fusion effect, a specially designed Layer Attention Fusion Block (LAFB) is introduced at the end of each scale stage. This module is capable of accurately performing channel-level alignment on the features output by the two pathways, eliminating the fusion barriers caused by differences in feature dimensions. Subsequently, it intelligently fuses the two types of features through dynamic weighting, automatically adjusting the weight ratio of the features from the two branches according to the expression requirements of defect features in different scenarios. This realizes the dual integration of spatial detail information and semantic abstraction information, thereby enabling the model to more comprehensively and accurately capture the complex features of irregular steel defects, significantly improving the model’s detection performance and environmental adaptability.

LAFB encompasses two core stages, namely hierarchical attention fusion and feature reconstruction and optimization, with its key component being the LocalGlobalAttention module. This component is designed to perceive local feature sources and incorporates a Parallelized Patch-aware Attention module to enhance feature perception. Specifically, the input to LAFB is a feature map $F_{1}\in R^{H\times W\times C_{1}}$, where H, W, and C_1_ represent the height, width, and number of channels of the input feature map, respectively, and the output is a feature map $R^{H\times W\times C_{2}}$ with the same spatial dimensions but an adjusted number of channels C_2_. Prior to the fusion process, the input feature map undergoes dimensionality reduction via 1 × 1 convolution to generate two intermediate feature maps F_1_ and F_2_; the fusion is performed in accordance with the following formulas:

(1) $$F_{\mathrm{base}}=C{\mathrm{onv}}_{3\times 3}(C{\mathrm{onv}}_{1\times 1}(F_{1})+C{\mathrm{onv}}_{1\times 1}(F_{2}))$$

In the design of the fusion pathway, the LocalGlobalAttention branch achieves complementary perception of fine-grained local information and global information by capturing features with different patch sizes. After a series of feature enhancement operations including attention weighting, cross-scale interaction, and nonlinear transformation, the enhanced feature map is obtained, which is expressed as

(2) $$X_{\mathrm{patch}}=Reshape(F_{base})\in R^{\frac{H}{P}\times \frac{W}{P}\times P^{2}\times C}$$

For every individual patch, after processing the local information of each feature patch layer by layer, probability-based attention weights are generated through SoftMax activation:

(3) $${z=\mathrm{Softmax}(\mathrm{MLP}}_{2}{(\mathrm{Norm}(\mathrm{MLP}}_{1}{(\mathrm{Mean}(X}_{\mathrm{patch}})))))$$

The Reshape operation is performed to restructure the dimensions of the weight features. Subsequently, the bilinear interpolation (BilinearInterp) method is adopted to restore the restructured feature matrix to the same spatial dimensions as the input feature map F1, ensuring the alignment of features in the spatial dimension and avoiding spatial information misalignment during the subsequent fusion process.

(4) $$F_{\mathrm{attn}}=\mathrm{BilinearInterp}(\mathrm{Reshape}(z⊙z))(k=1,2,3,4;\;P\in \left\{2,4\}\right)$$

Finally, four-branch features are concatenated with Fbase, undergoing 1 × 1 convolution, RepConv, and 1 × 1 convolution for fusion optimization:

(5) $$F_{\mathrm{out}}={\mathrm{Conv}}_{1\times 1}{(\mathrm{RepConv}}_{3\times 3}{(\mathrm{Conv}}_{1\times 1}([\sum_{k=1}^{4}F_{\mathrm{attn}}^{k}+F_{\mathrm{base}}])))$$

The LAFB relies on a parallel multi-branch structure, modeling fine-grained local textures and global contextual information for different patch scales respectively. Through token-wise normalization, adaptive dimensionality adjustment, and SoftMax attention weighting, it accurately quantifies the contribution weights of features across various scales. This effectively addresses the long-standing challenge in traditional fusion methods where local structural information and global semantic information are difficult to synergize, strongly ensuring the efficient deployment and reliable operation of the model on resource-constrained platforms.

### 3.3. Hierarchical Scale-Based Pyramid Attention Networks

In the steel defect detection scenario, defect targets often face significant scale variation challenges. On one hand, inherent size differences exist among different types of steel defects. On the other hand, variations in shooting distance and magnification of imaging equipment in industrial detection lead to drastically different scale ranges of the same type of defect in images, which poses great difficulties for the model to accurately capture defect features. Meanwhile, the visual features of some steel defects are relatively weak and easily confused with background textures, further increasing the detection difficulty.

**Figure 4:**
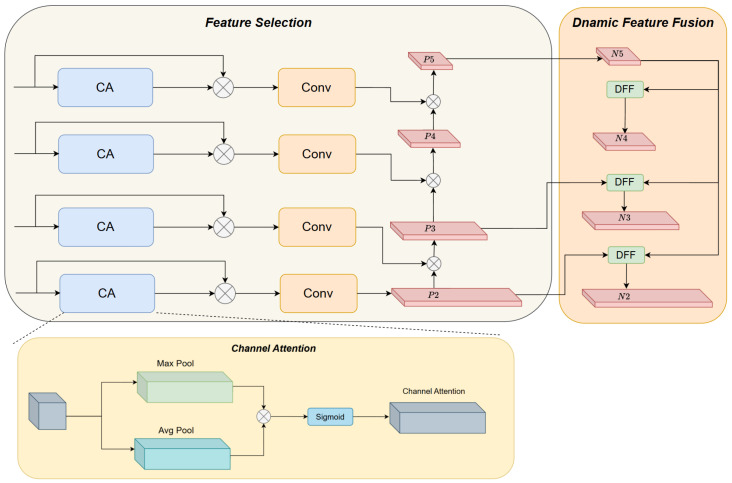
Structure of HSPAN.

To address the aforementioned issues, this paper designs a Hierarchical Scale-based Pyramid Attention Network (HSPAN). By means of selective fusion of multi-scale features, it enhances the model’s capability to represent features of steel defects across different scales. Composed of two components, namely Feature Selection module and Dynamic Feature Fusion module, as illustrated in Figure 4, the core idea of this module is to leverage the strong semantic information of high-level features to guide the screening of low-level features, thereby achieving accurate fusion of semantic information and detailed information.

**Figure 5:**
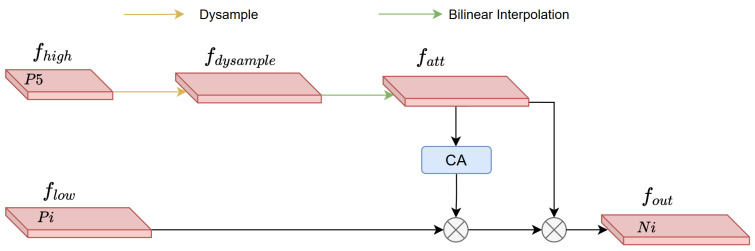
Structure of DFF.

Traditional multi-scale fusion methods often adopt simple pixel-wise addition to fuse high-level and low-level features. However, this approach fails to consider feature validity screening, making it prone to introducing useless information that interferes with the expression of defect features. To address this issue, this paper designs a Dynamic Feature Fusion (DFF) module, which achieves precise enhancement of low-level features under the guidance of high-level features. The specific process is as follows in Figure 5. For the input high-level feature F_high_ ∈ R^C×H×W^ and low-level feature F_low_ ∈ R^C×H1×W1^, the high-level feature is first subjected to scale expansion using a dysample upsampling module, resulting in F_high_ ∈ R^C×2H×2W^. The dysample upsampling module adaptively adjusts feature mapping through learnable parameters, enabling it to preserve the integrity of high-level semantic information while enlarging the feature map size. Bilinear interpolation is then used to further adjust the scale of the expanded high-level feature, yielding an attention feature F_att_ ∈ R^C×H1×W1^ that matches the size of the low-level feature, ensuring alignment in the spatial dimension. Fatt is fed into the Channel Attention (CA) module to generate attention weights, which are used to screen the low-level feature, highlighting detailed information related to steel defects in the low-level feature. Finally, the screened low-level feature is added point-by-point to the attention feature Fatt, obtaining the final fused feature F_out_ ∈ R^C×H1×W1^.

The fusion process not only injects strong semantic information into the low-level feature but also retains precise defect location details, significantly improving the model’s ability to recognize steel defects of different scales. The fusion process is expressed by the following formulas:

(6) $$F_{\mathrm{att}}=\mathrm{BilinearInterp}(\mathrm{Dysample}(F_{\mathrm{high}}))$$

(7) $$F_{\mathrm{out}}=F_{\mathrm{low}}*{\mathrm{CA}(F}_{\mathrm{att}})+F_{\mathrm{att}}$$

Compared with a single bilinear interpolation method, the combination of the dysample upsampling module and bilinear interpolation can more flexibly address the feature distortion problem during scale conversion. It adapts to the complex morphologies of steel defects through learnable parameters, providing a more reliable foundation for subsequent feature fusion.

### 3.4. OAHead

**Figure 6:**
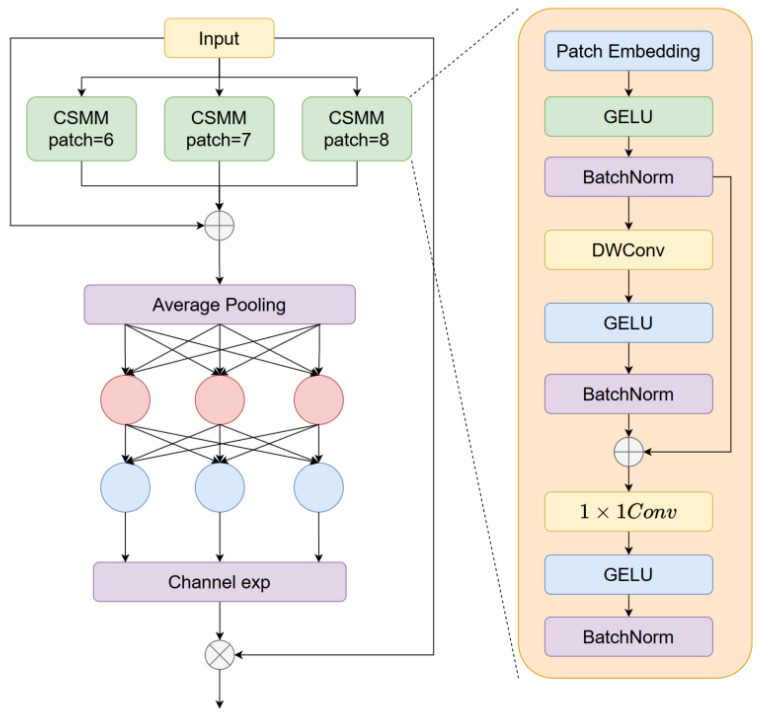
Structure of OAHead.

To tackle the problem that defects on steel surfaces may be covered by oxide scales, oil stains, or impurities, or mutually occluded owing to the dense distribution of multiple defects, thereby causing partial loss of defect features and blurred boundaries, which renders it challenging for the model to extract complete and valid features for precise localization and identification, an Occlusion-aware Attention Module, as illustrated in Figure 6, is introduced herein. By means of the dual effects of feature enhancement and loss constraint, the capability of the model to detect occluded steel defects is enhanced.

The core of the Channel Spatial Mixing Module (CSMM) is composed of depthwise separable convolution and a cross-channel fusion network, with residual connections and GELU activation function incorporated to mitigate gradient vanishing. Depthwise separable convolution adopts a channel-wise convolution approach to learn defect features within each channel individually; this reduces the number of model parameters while enabling precise capture of local valid information within single channels. However, depthwise separable convolution suffers from the issue of inter-channel information isolation, failing to leverage correlated features across different channels to compensate for information loss in occluded regions. To address this, 1 × 1 convolution is employed to fuse the output features of each channel, establishing inter-channel information interaction and preliminarily alleviating the problem of incomplete features caused by occlusion.

Subsequently, a two-layer fully connected network is employed to perform deep aggregation of the fused features, thereby further exploring the potential correlations among channels and strengthening the feature relevance between occluded and non-occluded regions. The output of the fully connected network undergoes exponential normalization, expanding the feature value range from [0, 1] to [1, e] and constructing a monotonic mapping relationship. This not only enhances the model’s tolerance to defect position offsets but also adapts to the problem of defect feature misalignment caused by occlusion. Finally, the generated attention weights are element-wise multiplied by the original feature map: effective feature responses in non-occluded regions are strengthened through weight assignment, while adaptive compensation is implemented for features in occluded regions, which significantly improves the model’s feature expression capability for occluded defects.

## 4. Experiment

In this section, the NEU-DET benchmark dataset, widely used in the field of metal surface defect detection, is adopted for experimental validation. First, with YOLOv12 [30] as the baseline model, a series of ablation experiments are designed and conducted to analyze the contribution of each proposed module to model performance, thereby verifying the effectiveness of the proposed scheme. Second, the network we proposed is compared with state-of-the-art models in the field, and the comprehensive efficiency of the proposed method is evaluated from the dimensions of precision, recall, and parameter count. Finally, through visual comparison, the advantages of the proposed model in localization and recognition performance for steel defect detection tasks are intuitively demonstrated.

### 4.1. Experimental Settings

In the experiment, the hardware configuration comprised an AMD Ryzen 7 5800X@3.4 GHz and an NVIDIA GeForce RTX 3080ti GPU. The model was trained on PyTorch 2.6.0 with acceleration enabled by CUDA 12.4, and the detailed hyperparameter configurations for the training process are tabulated in Table 1.

### 4.2. Dataset Description

This section employs NEU-DET [31], a benchmark dataset for metallic surface defect detection. First, a series of ablation experiments are conducted to verify the effectiveness of the proposed innovative scheme. Subsequently, a comparative analysis is carried out against recent state-of-the-art models to evaluate its efficiency, followed by visual comparison.

**Figure 7:**
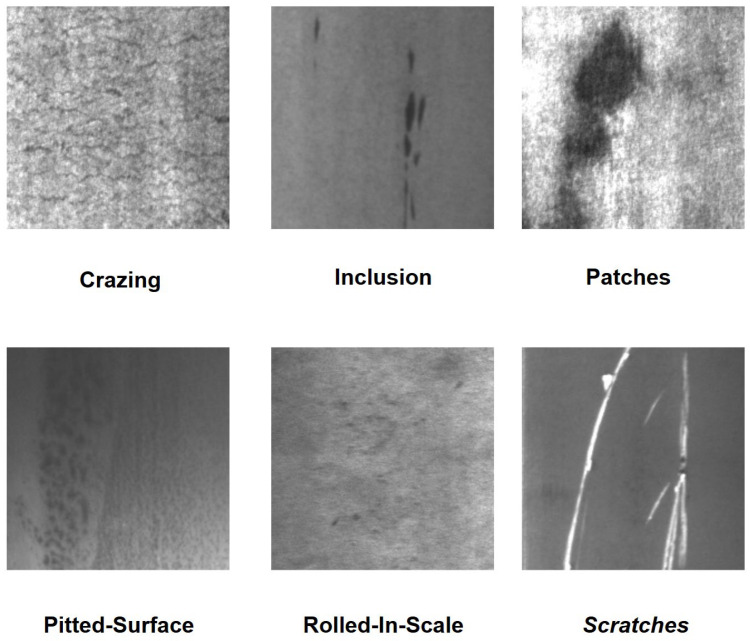
Example of NEU-DET Dataset.

To comprehensively verify the effectiveness of the proposed method in actual production line environments, this study adopts the open-source NEU-DET dataset. This dataset is constructed by Northeastern University and is dedicated to steel surface defect detection. It includes six common types of steel surface defects, namely Scratches (SC), Crazing (CR), Patches (PA), Inclusion (IN), Pitted Surface (PS) and Rolled-in Scale (RS). The dataset consists of 1800 grayscale images with a size of 200 × 200 pixels. The six typical types of steel surface defects mentioned above are illustrated in Figure 7. The experiment divides the dataset into a training set of 1440 images, a validation set of 180 images and a test set of 180 images following the ratio of 8:1:1.

**Figure 8:**
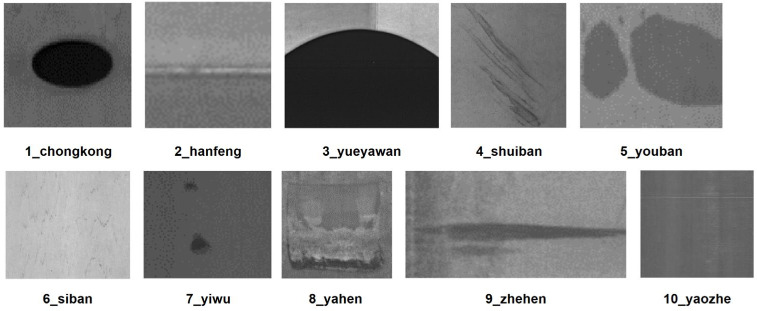
Example of GC-10 Dataset.

To verify the generalization ability of the model, this study additionally adopted the open-source GC-10 dataset [32]. It includes 10 common types of steel surface defects, namely Punching_hole(1_chongkong), Welding_line(2_hanfeng), Crescent gap(3_yueyawan), Water spot(4_shuiban), Oil spot(5_youban), Silk spot(6_siban), Inclusion(7_yiwu), Rolled pit(8_yahen), Crease(9_zhehen), and Waist folding(10_yaozhe). The ten typical steel surface defect types mentioned above are shown in Figure 8. The experiment divided the dataset into a training set (2856 images), a validation set (357 images), and a test set (357 images) in an 8:1:1 ratio.

**Figure 9:**
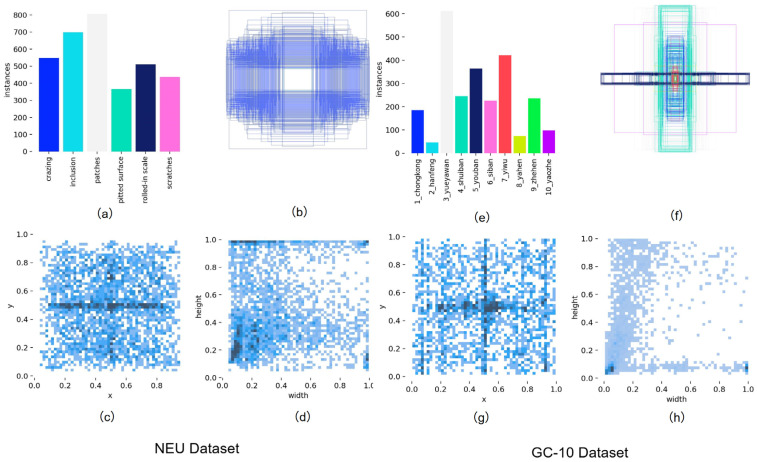
Distribution of the dataset. (**a**,**e**) Number of instances per defect category. (**b**,**f**) Bounding box scale distribution. (**c**,**g**) Bounding box center spatial distribution. (**d**,**h**) Bounding box aspect ratio distribution.

The detailed characteristics of the NEU-DET and GC-10 datasets can be systematically analyzed through the four dimensions depicted in Figure 9: (a,e) counts the number of annotated instances corresponding to each defect type, intuitively presenting the sample distribution of different defect categories in the dataset; (b,f) depicts the scale distribution characteristics of target bounding boxes, reflecting the overall distribution pattern of target sizes in the dataset; (c,g) shows the spatial distribution of the center point coordinates of bounding boxes, and the dense distribution feature indicates that the targets in this dataset are mainly of small to medium scales; and (d,h) is a scatter visualization of the aspect ratio of bounding boxes, where the darker color aggregation in the lower left area clearly reflects the distribution preference of bounding boxes in the width and height dimensions. Collectively, these features provide data distribution-level support for the subsequent training and performance evaluation of the model.

### 4.3. Evaluation Metrics

To evaluate model performance more intuitively, quantifiable indicators are used to compare the differences between different models, as follows:

Precision (P) represents the proportion of true positives within the set of predicted positive samples, a metric that reflects how reliable a model’s positive predictions are. A high precision score implies that the model rarely yields false positive results, with the corresponding calculation formula shown below.

(8) $$P=\frac{TP}{TP+FP}\times 100\%$$

Recall (R) denotes the ratio of samples correctly identified as positive to all actual positive samples in the dataset. It quantifies the sensitivity of a detection model, where a high recall value indicates that the model rarely incurs false negative errors. The corresponding calculation formula is given as follows.

(9) $$R=\frac{TP}{TP+FN}\times 100\%$$

Average Precision (AP) denotes the mean value of precision calculated across a full range of recall thresholds. The corresponding formula is provided as follows.

(10) $$AP=\int_{0}^{1}P(R)dR$$

Mean Average Precision (mAP) is computed by first calculating the area under the Precision-Recall (P-R) curve for each category, then taking the arithmetic mean of the Average Precision (AP) values across all categories. Specifically, mAP@0.5 denotes that a detection result is regarded as a true positive when the Intersection over Union (IoU) is greater than or equal to 0.5. Additionally, mAP@0.5:0.95 refers to calculating the AP at a sequence of IoU thresholds from 0.5 to 0.95 with an interval of 0.05, and then averaging these AP values arithmetically. The corresponding formula is provided as follows.

(11) $$mAP=\frac{1}{N}\sum_{i=1}^{N}AP_{i}$$

The F1-score is the harmonic mean of Precision and Recall, which comprehensively evaluates the detection performance by balancing the contributions of precision and recall. It ranges from 0 to 1, where a higher value indicates better overall performance with fewer false positives and false negatives.

(12) $$F1=2\times \frac{P\times R}{P+R}\times 100\%$$

In the aforementioned formulas, True Positive (TP) denotes the number of targets accurately detected by the model; False Positive (FP) refers to the count of predictions that are either misidentified by the model or correspond to non-existent targets; False Negative (FN) represents the quantity of real targets that the model fails to detect; and True Negative (TN) indicates the number of background regions where no targets exist and no spurious detections are generated.

Frames Per Second (FPS) quantifies the inference throughput of a model, defined as the number of complete image frames that the model can process within a one-second timeframe under fixed hardware and optimization conditions.

Additionally, to assess the model’s data efficiency and practical applicability in real-world scenarios, the number of parameters (Params) and floating-point operations per second (GFLOPS) were adopted as two key evaluation metrics in the experiments.

### 4.4. Comparison of Different Backbone

In this study, the control variable method was adopted to systematically evaluate the object detection performance of different backbone network modules on the NEU-DET dataset, as summarized in Table 2. The proposed LM Backbone exhibits pronounced advantages in lightweight design. Specifically, its parameter count is only 1.6 M, which is the smallest among all compared modules and represents a reduction of 0.9 M relative to the baseline. In addition, its computational complexity is reduced to 3.7 GFLOPs, corresponding to an approximately 36.2% decrease, thereby significantly outperforming HGNetV2 and Fasternet in terms of computational efficiency. Although the LM Backbone achieves slightly lower detection metrics, such as mAP@0.5, compared with the baseline and HGNetV2, it realizes substantial model compression and computational cost reduction while preserving fundamental detection capability. These results indicate that the LM Backbone better satisfies the core requirements of lightweight deployment, making it the most suitable backbone choice for resource-constrained terminal devices in this experiment.

### 4.5. Comparison of Different Pyrimid Network

The core principle for selecting the pyramid network in this study is guided by the computational constraints of practical industrial deployment; the detection model designed in this paper is targeted for deployment on industrial-grade low-computing-power embedded devices such as the NXP i.MX6ULL and Rockchip RK3568, which possess a floating-point computing capacity of only approximately 2–3 GFLOPS with extremely limited memory and computing resources. Therefore, maximally compressing the model’s parameter count and computational complexity while ensuring detection performance has become the primary criterion for pyramid network selection.

As shown in Table 3, although comparative pyramid networks such as BiFPN and HSFPN yield slightly higher detection metrics, the performance gap is within an acceptable range; the difference in mAP@0.5 does not exceed 1.3 percentage points. In contrast, the proposed HSPAN achieves the lowest computational complexity among all candidate networks while maintaining detection performance comparable to that of the comparative models, and its parameter count is on par with the lightest comparative models, making it the most suitable solution for deployment on low-computing-power embedded devices. Additionally, HSPAN is not merely a lightweight module; it is designed with dynamic feature selection and adaptive upsampling strategies tailored to the characteristics of industrial steel surface defect detection, such as large scale variations and weak visual features of defects. This enables it to effectively suppress industrial background noise and preserve defect detail information with low computing overhead. Thus, HSPAN achieves an optimal balance of detection performance, computing overhead and adaptability to industrial scenarios, and is therefore the final choice for this study.

### 4.6. Comparison of Different Head Detector

As shown in Table 4, although comparative detection heads such as EfficientHead, LQEHead, and LADH achieve competitive detection metrics on the NEU dataset, the performance gap is within a narrow range, with the difference in mAP@0.5 not exceeding 1.0 percentage point. While LADH and EfficientHead exhibit fewer parameters when used independently, experimental results demonstrate that their parameter counts both stabilize at 0.9 M when integrated with the proposed LM Backbone and HSPAN, failing to show any significant advantage. In contrast, the proposed OAHead achieves the highest mAP@0.5 and mAP@0.5:0.95 among all candidates while maintaining comparable parameter counts and computational complexity to the baseline, striking a better balance between performance and overhead. Furthermore, OAHead is not a generic detection head but is specifically designed to address the occlusion and feature incompleteness issues prevalent in steel surface defect detection, enabling it to effectively recover key defect features and suppress background interference without excessive computation. Thus, OAHead achieves an optimal trade-off among detection accuracy, computational efficiency, and industrial adaptability, making it the final choice for this study.

### 4.7. Multi-Fold Experiment for LHO-net

**Figure 10:**
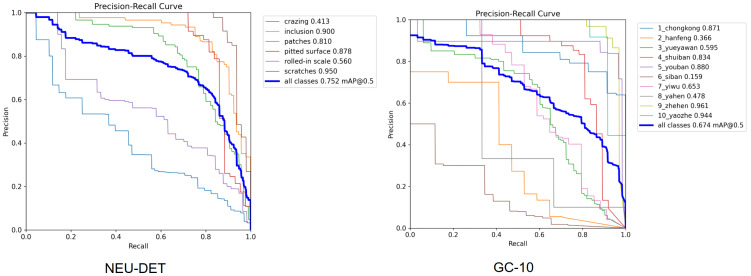
P–R curve of the proposed model on the NEU-DET and GC-10 dataset.

To verify the robustness of the proposed LHO-net, we adopted a multi-fold experimental scheme on both the NEU-DET and GC-10 datasets: the images of each dataset were evenly divided into 10 subsets, with 8 randomly selected as the training set, 1 as the validation set, and 1 as the test set. This process was repeated three times to obtain three independent experimental results. As shown in Table 5, the proposed model exhibits minimal fluctuations in all metrics on both datasets, with standard deviations controlled within 0.3%, demonstrating high stability and generalization ability. Therefore, in the subsequent performance comparison and analysis of the paper, all experimental data for the proposed LHO-net architecture are reported as the average of the three-fold experiments to ensure the objectivity and reproducibility of the evaluation. Meanwhile, Figure 10 presents the PR curve of the best-performing fold among the three experiments, which intuitively reflects the upper limit of the model’s detection performance under the optimal data split.

### 4.8. Ablation Experiment for LHO-net

To further analyze the collaborative behavior of the proposed modules, additional attention is given to Models D and F in Table 6 on the NEU-DET dataset. Model D integrates the LM Backbone and HSPAN modules while excluding the detection head optimization. Owing to the joint effect of lightweight feature extraction and efficient multi-scale fusion, Model D achieves an extreme reduction in model complexity, with only 1.0 M parameters and 2.9 GFLOPs. However, the detection accuracy shows a noticeable decline, particularly in mAP@0.5:0.95, indicating that although backbone and pyramid optimization effectively compress the model, they are insufficient to fully preserve localization precision for complex and occluded defects. This result highlights the limitation of relying solely on lightweight feature extraction and fusion without adequate detection head enhancement.

In contrast, Model F integrates all three proposed modules. By introducing OAHead on top of the lightweight backbone and pyramid structure, Model F effectively compensates for the accuracy degradation observed in Model D, leading to a substantial recovery in detection performance. Specifically, Model F achieves an mAP@0.5:0.95 of 44.0% while further reducing the parameter count to 0.9 M and the computational complexity to 2.3 GFLOPs. Compared with the baseline, this corresponds to reductions of 64% in parameters and 60.3% in computational cost, while both precision and recall are maintained at a level comparable to or higher than the baseline. These results clearly demonstrate that the proposed detection head plays a critical role in balancing accuracy and efficiency and that the full integration of the three modules is essential for achieving a lightweight yet high-performance defect detection network suitable for industrial terminal deployment.

To verify the model’s generalization, ablation experiments were conducted on the GC-10 dataset, focusing on Models D and F in Table 7. Model D combines the LM Backbone and HSPAN, achieving notable reductions to 1.0 M parameters and 2.9 GFLOPs via lightweight feature extraction and multi-scale fusion. However, its detection accuracy, especially mAP@0.5:0.95, decreased noticeably, revealing the limitation of lacking detection head enhancement for complex and occluded defects.

Model F integrates all three proposed modules, and the introduction of OAHead effectively mitigates the accuracy drop of Model D. It achieves 35.7% mAP@0.5:0.95, with parameters further reduced to 0.9 M and computational complexity to 2.3 GFLOPs. Compared with the baseline Model A, parameters and computational cost are reduced by 64% and 61.7% respectively, while precision and recall remain competitive. These results confirm that OAHead is critical for balancing accuracy and efficiency, and the full integration of the three modules ensures the model’s lightweight and high performance for industrial edge deployment.

### 4.9. Comparison with Different Models

As shown in Table 8, the proposed method is compared with several representative lightweight object detection models on the NEU-DET dataset. Traditional lightweight models such as YOLOv3-tiny exhibit limited detection accuracy, with mAP@0.5:0.95 of only 30.2%, indicating insufficient feature representation capability for complex defect scenarios. More recent lightweight variants, including YOLOv5n, YOLOv8n, YOLOv10n, and YOLOv11n, achieve improved detection performance but still require relatively higher computational cost and parameter scale. In contrast, the proposed method attains an mAP@0.5:0.95 of 44.0%, which is comparable to or even higher than most state-of-the-art lightweight YOLO variants, while significantly reducing model complexity. Specifically, the proposed model contains only 0.9 M parameters and requires merely 2.3 GFLOPs, achieving reductions of more than 60% in both parameters and computational cost compared with YOLOv5n and YOLOv12n. Although some models, such as YOLOv8n, achieve slightly higher mAP values, they rely on substantially larger model sizes and computational overhead. These results demonstrate that the proposed method achieves a superior trade-off between detection accuracy and efficiency, making it particularly suitable for real-time steel defect detection and deployment on resource-constrained industrial terminal devices.

As shown in Table 9, the proposed method is compared with several representative lightweight object detection models on the GC-10 dataset. To ensure fair evaluation on the GC-10 dataset, LHO-net was re-trained from scratch with the same hyperparameter settings as on the NEU-DET dataset, without leveraging any pre-trained weights from other datasets. Traditional lightweight models such as YOLOv3-tiny exhibit limited detection accuracy, with an mAP@0.5:0.95 of only 28.8%, indicating insufficient feature representation capability for the diverse and complex steel defect scenarios in the GC-10 dataset. More recent lightweight YOLO variants have improved detection performance compared to traditional models but still suffer from relatively high computational costs and large parameter scales, making them difficult to adapt to deployment requirements on resource-constrained industrial terminal devices. In contrast, the proposed method achieves an mAP@0.5:0.95 of 35.7%, which is comparable to or even higher than that of most state-of-the-art lightweight YOLO variants, while significantly reducing model complexity. Specifically, the proposed model contains only 0.9 M parameters and requires merely 2.3 GFLOPs, achieving reductions of more than 60% in both parameters and computational cost compared with baseline models such as YOLOv5n and YOLOv12n. Although some models like YOLOv8n achieve slightly higher mAP values, they rely on substantially larger model sizes and computational overhead as support. In summary, the proposed method achieves a superior trade-off between detection accuracy and efficiency, making it particularly suitable for resource-constrained industrial terminal devices and capable of meeting the practical deployment requirements of real-time steel defect detection.

### 4.10. Visualization Results

**Figure 11:**
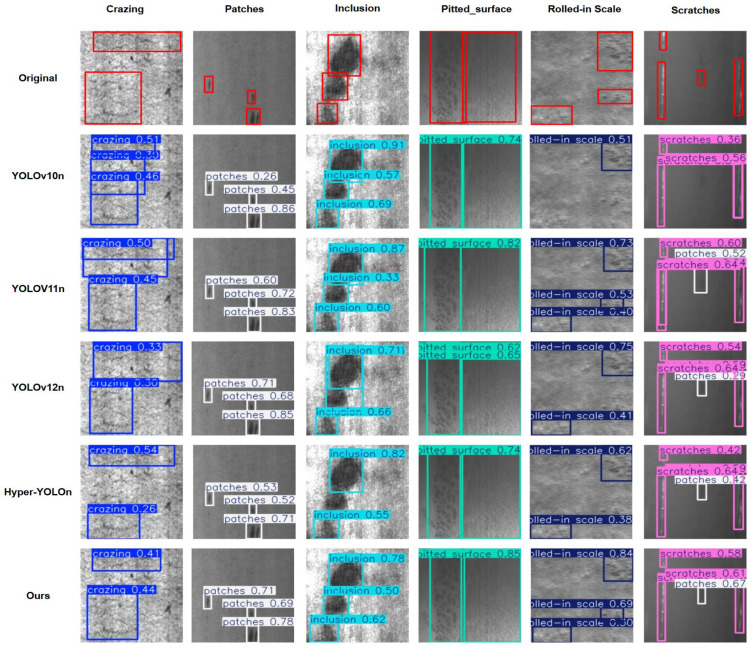
Detection results of different models on the NEU-DET dataset.

The visualization experiment results are shown in Figure 11. From the visualization outcomes, distinct differences in false detection and missed detection are observed among different models in the detection of 6 types of steel defects. YOLOv10n presents redundant multi-box annotations for “Inclusion”, and its confidence score for “Rolled-in_Scale” is only 0.51, indicating a risk of missed detection. YOLOv11n has false detections in the “Scratches” category; meanwhile, the confidence scores of some boxes for “Inclusion” are lower than 0.6, showing insufficient ability to recognize small-scale inclusions. Although YOLOv12n has a high confidence score for “Patches”, its confidence score for “Rolled-in_Scale” fluctuates greatly, leading to poor robustness for defects with weak features. Hyper-YOLOn has repeated box annotations in the “Crazing” category, and its low confidence score for “Scratches” makes it prone to missing slender scratch defects. In contrast, the model proposed in this study not only avoids the issues of redundant multi-boxes and false detections existing in other models but also stably recognizes various defects: for example, its confidence score for “Rolled-in_Scale” remains above 0.6, and both the framing accuracy and confidence score for “Scratches” are better than those of the comparison models. At the same time, it does not miss the annotation of small-scale “Inclusion”. These results fully demonstrate the advantages of its multi-scale feature fusion and occlusion-aware modules in reducing false/missed detections and improving detection stability.

This result validates the effectiveness of the HSPAN multi-scale feature fusion and OAHead occlusion-aware modules in the model: the former enhances the feature transmission of defects across different scales, while the latter compensates for feature loss in occluded or blurred scenarios. Ultimately, this enables our model to achieve accurate recognition of various steel defects while maintaining extreme light weight, making it well-suited for practical detection requirements in industrial terminal environments.

**Figure 12:**
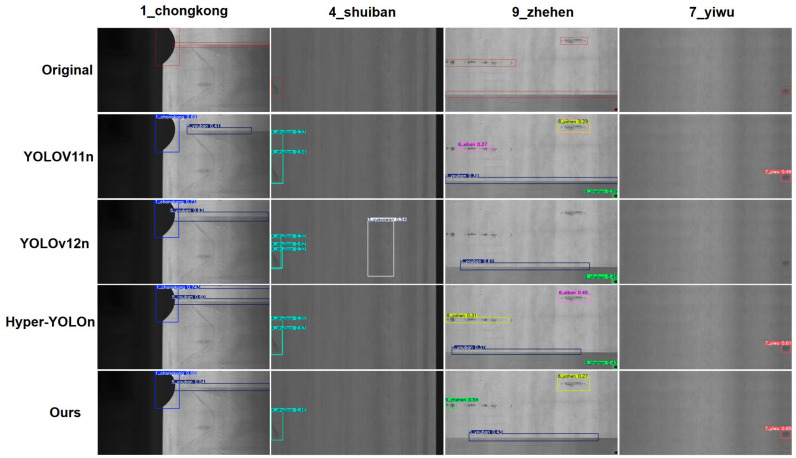
Detection results of different models on the GC-10 dataset.

The visualization experiment results are shown in Figure 12. From the outcomes of defect detection on the GC-10 dataset, clear discrepancies in false detection, missed detection, and positioning accuracy are observed among different models. YOLOv11n has low confidence scores for “9_zhehen” and incomplete positioning of slender crease defects; YOLOv12n produces redundant detection boxes for “4_shuiban” and fluctuates in confidence for occluded “7_yiwu”; and Hyper-YOLOn has repeated annotations for “9_zhehen” and insufficient positioning accuracy for small-scale defects. In contrast, the model proposed in this study avoids these issues; it achieves precise positioning for “1_chongkong” with stable confidence, effectively suppresses background interference to eliminate misdetection of “4_shuiban”, fully covers slender “9_zhehen” defects with high confidence, and accurately identifies occluded “7_yiwu” without missing annotations. These results fully demonstrate the advantages of our model in improving positioning accuracy, reducing false/missed detections, and enhancing robustness to complex defect scenarios.

This result validates the effectiveness of the key modules in our model: the lightweight dual-branch backbone ensures accurate capture of defect shape and texture features while maintaining low computational overhead; the dynamic feature selection mechanism strengthens the perception of low-contrast defects; and the occlusion-aware detection head compensates for feature loss in occluded scenarios. These enable our model to achieve reliable detection of diverse steel defects on the GC-10 dataset, making it suitable for practical industrial terminal deployment.

## 5. Conclusions

To advance the industrial application of steel surface defect detection technology, this study proposes and validates a novel detection network, LHO-net. Addressing the limitations of existing methods in adapting to complex scenarios and feasibility of terminal deployment, innovative improvements are made in three core directions, feature extraction, feature fusion, and occluded defect optimization, achieving remarkable results.

In terms of feature extraction, the LM Backbone adopts a dual-branch structure with shared HGStem and a dynamic feature fusion mechanism. While extremely compressing parameters and computational complexity, it effectively captures multi-dimensional features of irregular defects. In the feature fusion stage, the HSPAN realizes efficient fusion of multi-scale features through dynamic selection and adaptive upsampling, balancing noise suppression and detail preservation. The OAHead compensates for feature loss in occluded regions through deep feature aggregation and exponential normalization, enhancing the ability to recognize complex defects. Experimental results show that LHO-net achieves 75.0% mAP@0.5, 44.0% mAP@0.5:0.95, and 73.6% recall on the NEU-DET dataset, and mailto:67.1%25mAP@0.5, 67.2% mAP@0.5, 35.7% mAP@0.5:0.95 on the GC-10 dataset, with only 0.9 M parameters and 2.3 GFLOPS computational complexity—reductions of 64% and 60.3% compared to the baseline, respectively. Its lightweight advantage is significantly superior to mainstream YOLO series models.

Despite LHO-net’s breakthrough in balancing performance and lightweight design, the model still has certain limitations in practical industrial applications. Specifically, LHO-net exhibits relatively decreased detection accuracy for ultra-small steel defects (pixel size < 10 × 10) and defects with extremely low contrast against the background, as the lightweight feature extraction structure inevitably leads to partial loss of fine-grained detail information. Typical failure cases observed in the experiments include missed detections of micro-inclusions on the NEU-DET dataset and false detections of low-contrast water spots on the GC-10 dataset, which are mainly caused by the insufficient retention of tiny defect texture features and the weak discrimination of low-contrast defect regions from background noise under the lightweight design constraint. In addition, a comparative discussion with closely related lightweight steel defect detection studies further reveals the model’s characteristics: compared with state-of-the-art methods such as YOLOv12n and Hyper-YOLOn, LHO-net shows obvious advantages in extreme parameter compression and adaptability for edge device deployment, but there is a certain gap in the detection performance of ultra-small and low-contrast defects, which is the key direction for subsequent optimization.

Despite LHO-net’s breakthrough in balancing performance and lightweight design, the complexity of industrial scenarios, multi-environment adaptation requirements, and real-time deployment efficiency pose higher demands. Future work will expand the model’s generalization ability to adapt to different production environments and defect morphologies; deepen lightweight design to reduce hardware dependence; and adopt TensorRT technology for quantitative acceleration, deploying the optimized model to edge devices such as NXP i.MX6ULL to achieve industrial implementation.

## Figures and Tables

**Table 1:** Specific parameter settings in the model training process.

Training Parameters	Details
Epochs	300
Image Size	640 × 640
Batch Size	8
Workers	0
Initial Learning Rate	0.01
Final Learning Rate	0.001
Momentum	0.937
Mosaic	1.0
IoU ratio	0.7
Optimization Algorithm	SGD

**Table 2:** Comparison of different backbone on the NEU dataset.

Module	P (%)	R (%)	F1	mAP@0.5 (%)	mAP@0.5:0.95 (%)	Params	GFLOPS
No replacement	69.4	71.0	70.2	76.0	43.1	2.5 M	6.0
HGNetV2 [33]	69.2	**75.4**	72.2	**77.7**	42.5	2.1 M	5.5
Fasternet [34]	**71.7**	72.8	**72.8**	76.3	**45.3**	3.9 M	9.2
DAF [35]	71.2	70.5	70.8	73.3	42.8	4.5 M	9.4
LM Backbone	70.8	71.3	71.1	73.4	42.6	**1.6** M	**3.7**

**Table 3:** Comparison of different Pyrimid Networks on the NEU dataset.

Module	P (%)	R (%)	F1	mAP@0.5 (%)	mAP@0.5:0.95 (%)	Params	GFLOPS
No replacement	69.4	71.0	70.2	76.0	43.1	2.5 M	6.0
HSFPN [25]	70.7	71.2	71.0	75.7	43.5	1.8 M	5.3
EMBSFPN [36]	**71.3**	**72.7**	**72.0**	**75.9**	**42.9**	2.1 M	6.3
BiFPN [37]	69.4	70.8	70.1	76.9	42.9	1.9 M	6.0
HSPAN	70.6	72.2	71.4	75.6	41.8	**1.8** M	**5.0**

**Table 4:** Comparison of different Head Detectors on the NEU dataset.

Module	P (%)	R (%)	F1	mAP@0.5 (%)	mAP@0.5:0.95 (%)	Params	GFLOPS
No replacement	69.4	71.0	70.2	76.0	43.1	2.5 M	6.0
EfficientHead [38]	70.6	**73.3**	71.9	77.1	43.9	**2.2** M	**4.6**
LQEHead [39]	74.5	72.3	**73.4**	77.7	44.3	2.5 M	6.0
LADH [21]	74.3	70.1	72.1	78.0	44.6	2.2 M	4.7
OAHead	**74.6**	72.2	**73.4**	**78.1**	**44.8**	2.4 M	5.3

**Table 5:** Multi-fold Experiment for LHO-net on different datasets.

Dataset	P (%)	R (%)	F1 (%)	mAP@0.5 (%)	mAP@0.5:0.95 (%)
NEU-DET	70.9	73.8	72.32	74.9	44.0
	71.6	73.6	72.58	75.2	44.1
	71.2	73.3	72.24	75.0	44.0
Average	71.3 ± 0.3	73.6 ± 0.3	72.38 ± 0.2	75.0 ± 0.2	44.0 ± 0.1
GC-10	66.0	67.1	66.54	67.4	35.8
	65.7	67.2	66.44	67.1	35.6
	65.8	67.0	66.40	67.2	35.7
Average	65.8 ± 0.3	67.1 ± 0.1	66.46 ± 0.08	67.2 ± 0.2	35.7 ± 0.1

**Table 6:** Ablation Experiment for LHO-net on the NEU dataset.

Model	LM Backbone	HSPAN	OAHead	P	R	F1	mAP@0.5	mAP@0.5:0.95	Params	GFLOPS
A	-	-	-	69.4	71.0	70.2	76.0	43.1	2.5 M	6.0
B	√	-	-	70.8	71.3	71.1	73.4	42.6	1.6 M	3.7
C	-	√	-	70.6	72.2	71.4	75.6	41.8	1.8 M	5.0
D	√	√	-	70.4	71.6	71.0	73.5	40.8	1.0 M	2.9
E	-	-	√	**74.6**	72.2	**73.4**	**78.1**	**44.8**	2.4 M	5.3
F	√	√	√	71.3	73.6	72.4	75.0	44.0	**0.9** M	**2.3**

Note: √ means that the module is used; - means that the module is not used.

**Table 7:** Ablation Experiment for LHO-net on the GC-10 dataset.

Model	LM Backbone	HSPAN	OAHead	P	R	F1	mAP@0.5	mAP@0.5:0.95	Params	GFLOPS
A	-	-	-	66.7	59.0	62.6	64.3	34.8	2.5 M	6.0
B	√	-	-	64.7	63.4	64.0	65.0	34.6	1.6 M	3.7
C	-	√	-	64.9	65.4	65.2	64.8	34.5	1.8 M	5.0
D	√	√	-	65.3	66.3	65.8	65.6	35.4	1.0 M	2.9
E	-	-	√	**67.1**	65.5	66.3	**69.2**	**36.6**	2.4 M	5.3
F	√	√	√	65.8	**67.1**	**66.5**	67.2	35.7	**0.9** M	**2.3**

Note: √ means that the module is used; - means that the module is not used.

**Table 8:** Comparison with different models on the NEU dataset.

Module	P (%)	R (%)	F1	mAP@0.5 (%)	mAP@0.5:0.95 (%)	Params	GFLOPS	FPS
YOLOv3-tiny [40]	55.5	71.9	62.7	63.8	30.2	12.1 M	18.9	89
YOLOv5n	72.1	71.4	71.7	77.3	42.5	2.5 M	7.1	**143**
YOLOv8n	72.4	74.8	**73.6**	**77.9**	**45.5**	3 M	8.1	128
YOLOv10n [41]	66.9	69.6	68.2	73.5	42.0	2.7 M	8.2	106
YOLO11n	69.1	**74.9**	71.9	76.8	43.8	2.6 M	6.3	120
YOLOv12n	69.4	71.0	70.2	76.0	43.1	2.5 M	6.0	108
Hyper-YOLOn [42]	**72.9**	74.2	73.5	76.9	44.1	3.6 M	9.5	98
Ours	71.3	73.6	72.4	75.0	44.0	**0.9** M	**2.3**	131

**Table 9:** Comparison with different models on the GC-10 dataset.

Module	P (%)	R (%)	F1	mAP@0.5 (%)	mAP@0.5:0.95 (%)	Params	GFLOPS	FPS
YOLOv3-tiny	50.9	55.8	53.2	51.7	28.8	12.1 M	18.9	78
YOLOv5n	62.3	60.3	61.3	64.7	34.4	2.5 M	7.1	114
YOLOv8n	57.4	70.3	63.2	64.9	33.5	3 M	8.1	105
YOLOv10n	60.9	64.1	62.5	66.0	**35.8**	2.7 M	8.2	110
YOLO11n	65.3	66.4	65.8	**67.6**	35.4	2.6 M	6.3	115
YOLOv12n	66.7	59.0	62.6	64.3	34.8	2.5 M	6.0	97
Hyper-YOLOn	63.3	58.4	60.8	64.8	34.6	3.6 M	9.5	90
Ours	**65.8**	**67.1**	**66.4**	67.2	35.7	**0.9** M	**2.3**	**118**

## Data Availability

The code has been open sourced on GitHub: https://github.com/bi11ychan/LHO-net.git, accessed on 16 March 2026.
